# Mixed Natural Gas Online Recognition Device Based on a Neural Network Algorithm Implemented by an FPGA

**DOI:** 10.3390/s19092090

**Published:** 2019-05-05

**Authors:** Tanghao Jia, Tianle Guo, Xuming Wang, Dan Zhao, Chang Wang, Zhicheng Zhang, Shaochong Lei, Weihua Liu, Hongzhong Liu, Xin Li

**Affiliations:** 1 Department of Microelectronics, School of Electronics and Information Engineering, Xi’an Jiaotong University, Xi’an 710049, China; jiatanghao@stu.xjtu.edu.cn (T.J.); gtl940801@stu.xjtu.edu.cn (T.G.); xmwang_zw@126.com (X.W.); zhaodan_xjtu@163.com (D.Z.); wangc254@163.com (C.W.); lwhua@mail.xjtu.edu.cn (W.L.); 2 State Key Laboratory for Manufacturing Systems Engineering, Xi’an Jiaotong University, Xi’an 710049, China; zhang.zc1113@stu.xjtu.edu.cn (Z.Z.); leisc@mail.xjtu.edu.cn (S.L.); hzliu@mail.xjtu.edu.cn (H.L.)

**Keywords:** mixed gas, recognition, neural network, FPGA

## Abstract

It is a daunting challenge to measure the concentration of each component in natural gas, because different components in mixed gas have cross-sensitivity for a single sensor. We have developed a mixed gas identification device based on a neural network algorithm, which can be used for the online detection of natural gas. The neural network technology is used to eliminate the cross-sensitivity of mixed gases to each sensor, in order to accurately recognize the concentrations of methane, ethane and propane, respectively. The neural network algorithm is implemented by a Field-Programmable Gate Array (FPGA) in the device, which has the advantages of small size and fast response. FPGAs take advantage of parallel computing and greatly speed up the computational process of neural networks. Within the range of 0–100% of methane, the test error for methane and heavy alkanes such as ethane and propane is less than 0.5%, and the response speed is several seconds.

## 1. Introduction

It is predicted that natural gas will soon become the largest energy resource in the world, surpassing petroleum and coal [1,2,3]. In the process of mining natural gas, the crude gas contains about 15 components. Methane, ethane and propane and other alkane gases are the main components of natural gas [4]. To ensure the quality of crude gas, we need to analyze the concentration of each component in it. Gas chromatography is the most commonly used method to determine total gas composition and each gas concentration range in natural gas [5,6,7,8]. The column chromatographic separation technique is based on the gas flow phase. The main basis for separation is that in the gas sample, different components have different adsorption degrees or solubility in the chromatographic column.

However, the overlapping of chromatography makes it difficult to distinguish some gases and components. In addition, it is difficult to use in an automatic online detection system, because of its non-electrical output signal. Most importantly, if the chromatography method is used, it takes several hours to perform measurements, which cannot satisfy requirements. The online system composed of gas sensors with electrical parameters is a promising development of great current interest [9,10,11,12].

Currently, many methods are used to measure the concentration of mixed gases, or to classify different gases based on the neural network (NN) technique. NN is a kind of computation technique which is widely used for image classification [13], image recognition [14], speech recognition [15] and multi-parameter recognition. Areej Shahid et al. used traditional semiconductor sensors array and NN to distinguish between methane and carbon monoxide so as to measure the concentration of both gases [16]. Pai Peng, Xiaojin Zhao et al. proposed a new deep convolutional NN to classify different gases [17]. Bartosz Szulczyński et al. used six TGS-type sensors and one PID-type sensor to recognize single odors in three-component mixtures [18]. An artificial neural network (ANN) can be obtained by training an initial NN with a characteristic mixture gas sample database. An ANN sensors array can clearly distinguish the components and the concentration values of the components in the mixture. Shoffi Izza Sabilla et al. used an MQ-type ANN sensors array to detect the existence of gas in the air [19]. Sharvari Deshmukh et al. designed an ANN sensors array to measure obnoxious odors emitted from the pulp and paper industries [20]. A. Szczurek et al. used an ANN sensors array of six TGS-type sensors to identify organic gases [21]. Guillaume Hudon et al. used an ANN sensors array to measure odor intensity [22].

There are also some ANN applications implemented by a Field-Programmable Gate Array (FPGA). De Souza et al. implemented an RBF neural network by an FPGA, which could be used in practical situations of greater complexity [23]. Gaikwad et al. implemented an MLP classifier to detect human activities [24]. Zeyad Aklah et al. presented a multilayer perceptron Co-processor (MLPCP) targeting FPGAs that is configurable during design time and programmable during run time [25]. Fayçal Benrekia et al. developed a primitive gas recognition system for discriminating between industrial gas species using multilayer perceptron implemented by an FPGA [26]. However, it is currently often reported that mixed gases must be separated into individual gases before they are input into an ANN sensors array, since the ANN sensor mentioned above can only recognize its type and concentration in a single gas. In fact, ANN is a multi-input and multi-output non-linear recognizing system. When ANN is trained by a large data sample, it can recognize the type of gases and their concentrations simultaneously in mixed gases. However, research work in this area is still relatively rare.

On the other hand, in order to apply NN technology in practical multi-parameter recognition, the recognition system must support the calculation of NN from both computation scale and computation speed. Only when the computation speed is fast enough can the real-time identification requirements be met. However, the computation scale and speed of existing NNs are far from the level of real-time testing. Therefore, it is imperative to develop the hardware implementation of the NN computer. An FPGA contains an array of programmable logic blocks, and can be reprogrammed to implement different logic functions, allowing flexible reconfigurable NN computing as performed in computer software. 

It is desirable to use a trained ANN sensors array to accurately recognize the concentration of each single gas in mixed gases rather than separating mixed gases into individual gases by the chromatographic column and related methods. In this paper, we propose a mixed gas identification device based on a back-propagation (BP) algorithm, which can be used for the online detection of natural gas. Infrared gas sensors are used to build a gas sensors array due to their advantages of rapid response, large measurement range, and long life. A multi-layer perceptron neural network (MLP NN) combines the sensor arrays, which are conducted to recognize each gas concentration and to eliminate the cross-sensitivity of multi-component gases. The NN algorithm is implemented by an FPGA in the device, which has the advantages of small size, high precision and fast response. We are especially concerned about fast response, the most important advantage of our system. The response time can reach about 30 seconds, which is much faster than the chromatography method.

## 2. Methodology

### 2.1. Sample Database Experiment

**Figure 1:**
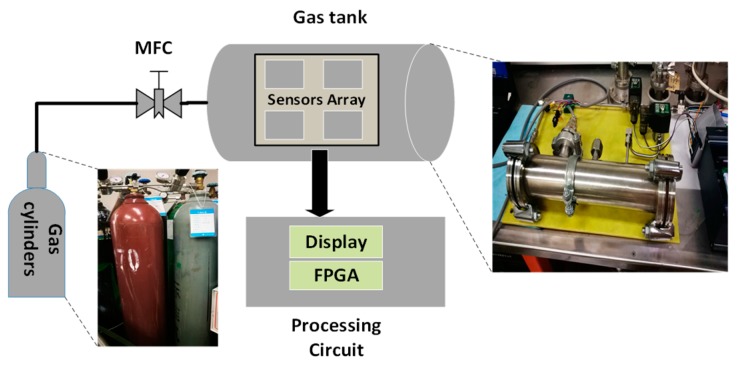
Schematic diagram of the structure of the mixed natural gas online testing system (the inserted pictures are the photos of the corresponding parts).

Our measurement system consists of three parts: gas pipeline system, sensors array system and processing circuit system, as shown in Figure 1. The pipeline system is composed of multi-channel high purity gas and its flow rate controller. The flow rate controller is produced by Beijing Sevenstar Electronics Corporation. The sensors array consists of three infrared gas sensors which are used to measure the concentrations of methane, ethane and propane, respectively, in the gas tank. The processing circuit consists of an FPGA chip, a liquid crystal display (LCD) and other circuit elements. The details of the sensors are shown in Table 1. The FPGA chip is used for receiving the data from the sensors and processing it using NN. The LCD is used to display the data processed by the FPGA.

### 2.2. Multilayer Layer Perceptron Neural Network (NN)

**Figure 2:**
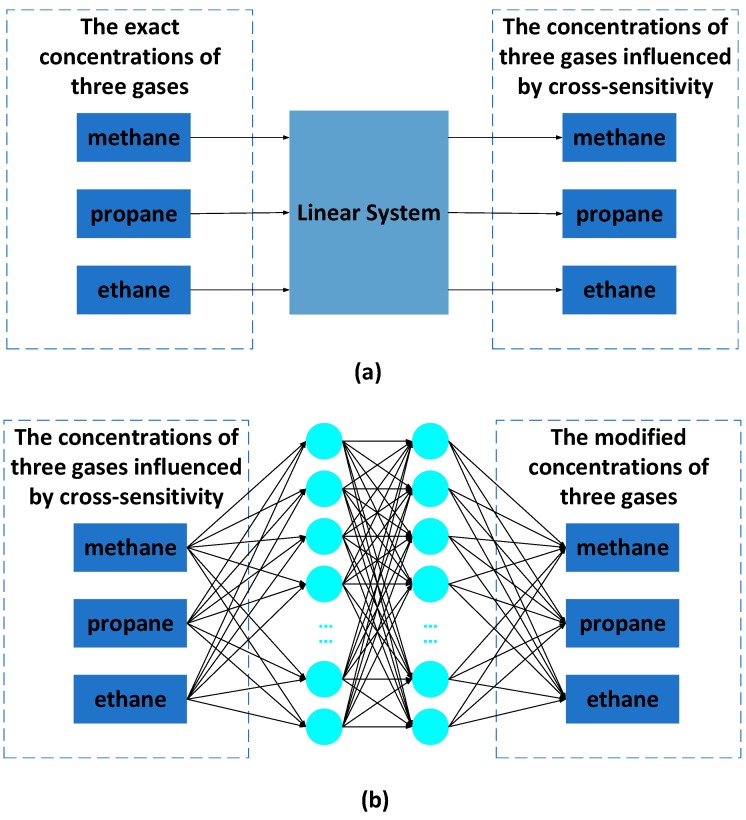
Two types of mixed gases recognition systems: (**a**) is a linear system, in which the outputs of the concentrations of the three gases are influenced by cross-sensitivity; (**b**) is a neural network (NN) recognition system, in which the outputs are close to the exact concentrations.

**Figure 3:**
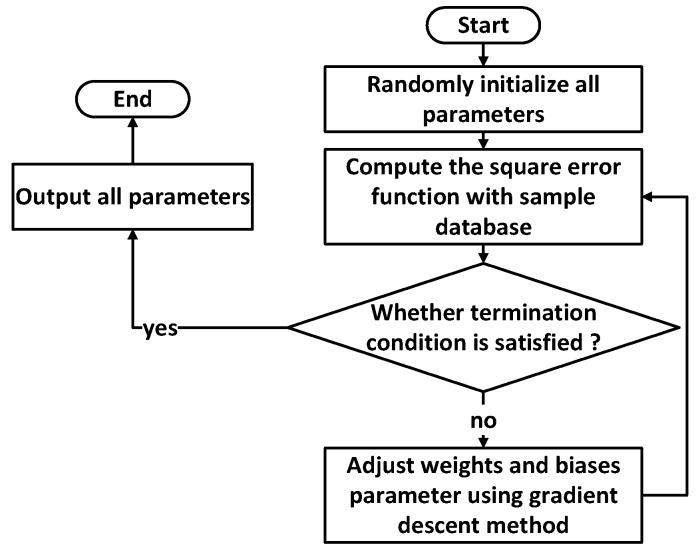
Flow chart of the NN training process using the gradient descent method.

A multilayer layer perceptron NN (MLP NN) can learn and store a large number of input–output mode mapping relations without revealing the mathematical equations describing the mapping relations beforehand. Through experimental testing, a large-scale dataset of three-input and three-output experimental samples can be constructed. Then the data sample library is used to train the NN to achieve the recognition accuracy required by the design. Our NN consists of one input layer, two hidden layers and one output layer. The input layer has 3 neurons; the first hidden layer has 10 neurons; the second hidden layer has 20 neurons; the output layer has 3 neurons. We use a back-propagation algorithm to train the MLP NN. MLP NN training consists of two processes: forward propagation of data flow and backward propagation of error signals. The direction of positive propagation is input layer, hidden layer and output layer, which is shown in Figure 2. The flow chart of the MLP NN training algorithm is shown in Figure 3, which describes the steps in the process of training the NN. The concrete steps of the process are described as follows:

The steps in the process of training the NN can be described with the flow chart in Figure 3.

1. Randomly initialize all parameters with small numbers, including weights and biases in each layer;
2. Calculate the value of error function, and compare it with the value at the output layer;
3. If the error function value does not satisfy the value at the output layer, adjust the parameters using the gradient descent method, then go back to step (2);
4. Otherwise, output all parameters and NN training is complete.

In order to train the MLP NN with strong generalization, a large database of representative samples needs to be prepared. Here, three kinds of gas with known concentrations were injected into the gas tank in turn, and the values of the three sensors were measured, so as to obtain a large range of samples for the learning and training database. 

The input layer in this work has 3 units. The hidden layer has bi-layers as the first hidden layer with 10 units, and the second hidden layer with 20 units. The output layer has 3 units.

### 2.3. Circuit Implementation

**Figure 4:**
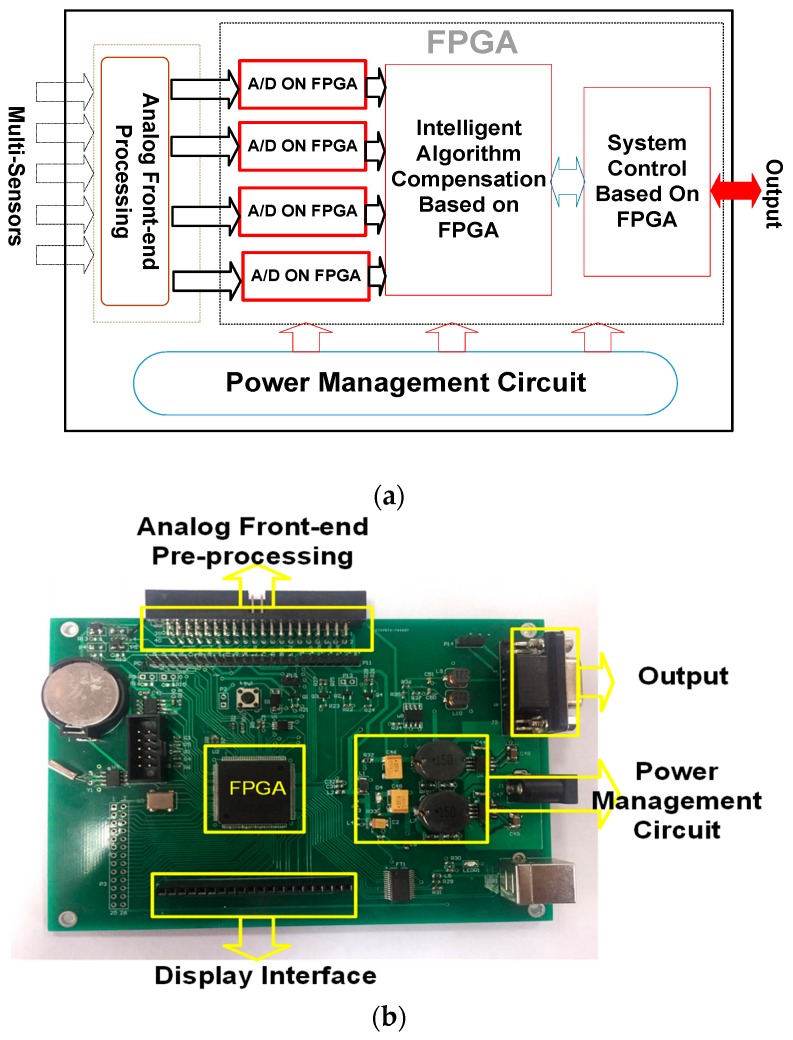
(**a**) Diagram of the circuit system framework of the mixed natural gas online testing system, in which the FPGA is the core of the intelligent algorithm and control module; (**b**) Processing circuit, in which the FPGA plays an important role. The FPGA is used to implement the NN algorithm and system control.

The framework of the circuit system is shown in Figure 4. The front-end multi-channel gas sensors (Multi-Sensors) detect the concentration of each gas and generate corresponding electrical signals for the analog front-end pre-processing. 

The sensor signal pre-processing module is used to process the signal from sensors and send to the on-chip analog-to-digital converter to acquire the digital signal for subsequent processing (A/D ON FPGA). Multiple power management circuits conduct management and provide multiple power interfaces for the operating voltages required by the different modules. The intelligent control module is used to control the operation of each module of the detection device, and performs real-time control according to different working conditions. The algorithm module, the processing chip, uses the corresponding algorithm to extract the characteristic response of the front-end acquisition signal, and outputs the desired useful signal; the liquid crystal display module processes and displays useful information that needs to be output; the RS485 interface module converts data into available communication signals for data transmission for remote data upload and maintenance.

**Figure 5:**
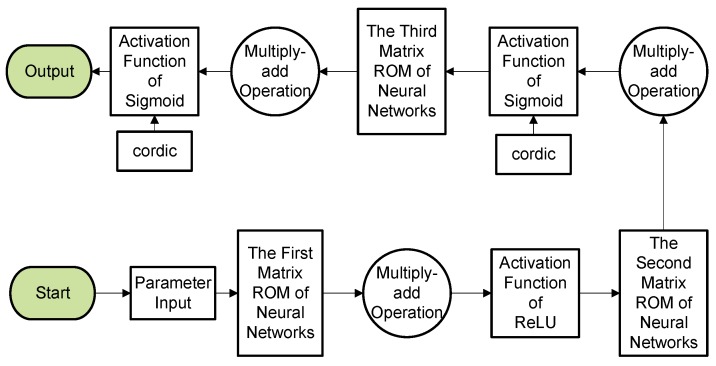
Flow chart of FPGA-based MLP NN implementation.

The Intelligent Learning Algorithm is the key module of the MLP NN algorithm implemented by hardware. Before the algorithm is conducted, the input signal of the sensor is converted to hexadecimal from a 32-bit floating-point. FPGAs contain an array of programmable logic blocks, and can be reprogrammed for different logic functions, allowing flexible reconfigurable NN computing as performed in computer software. The data obtained by the sensor is input from the input layer, then the parameters are extracted from the ROM to the conductor multiply add operation, and then input into the activation function. The subsequent processing of the hidden layer and the output layer is similar to that of the input layer. Figure 5 shows the flow chart of the algorithm, which mainly includes the implementation of the three-level matrix multiplication and activation function. Processing of the hidden layer and the output layer is similar to that of the input layer. The weight matrix parameters are stored in ROM. The analytic equations of the ReLU and Sigmoid functions are shown in Equations (1) and (2), respectively. The ReLU function could provide a fast training speed. The Sigmoid function has the advantage of high precision, but it has expansive computational costs. So, we combine the two functions to obtain best results.

(1) ReLU(x)=max(0,x)

(2) $$\mathrm{Sigmoid}\left(x\right)=\frac{1}{1+e^{-x}}$$

The expression “max (0, *x*)” means the maximum value of 0 and *x*.

## 3. Results and Discussion

**Figure 6:**
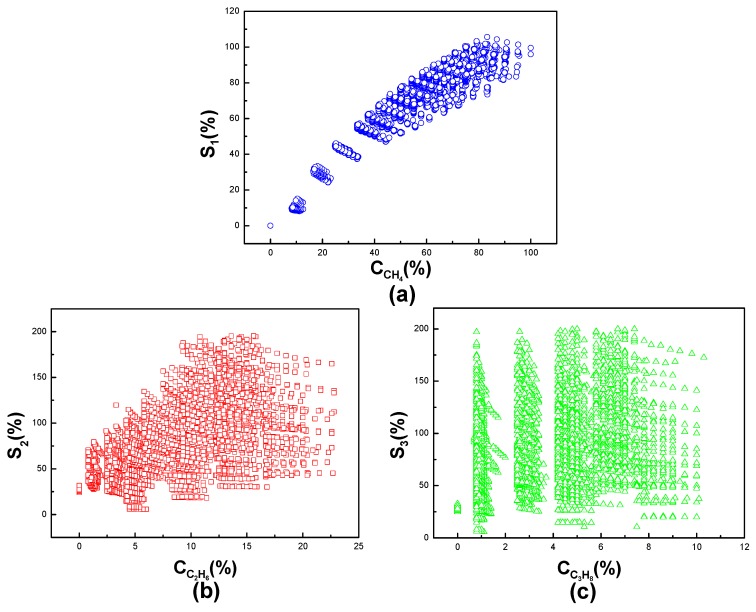
The output of the three gases obtained by the linear system. (**a**)–(**c**) shows the concentrations of methane, ethane and propane obtained in the linear system, respectively.

Because of the cross-sensitivity of the three kinds of gases to the sensors array, it is hardly possible to read the accurate concentration of each gas from linear system. The symbols used in the database are explained in Table 2, where $C_{\mathrm{in}}$ is the input concentrations of the three gases, $C_{\mathrm{sout}}$ is the sensors’ output of gases’ concentrations, and $C_{\mathrm{nout}}$ is the MLP NN value at the output layer, which is the concentrations of the three gases after eliminating cross-sensitivity. S_1_, S_2_ and S_3_ are the sensors’ output of methane, ethane and propane, respectively. The error represents the average error between sensors’ output and MLP NN output. Figure 6 shows the cross-sensitivity of the three sensors to the three kinds of gases before using MLP NN. The *X*-axis represents the accurate concentrations of the three gases methane, ethane, and propane, respectively. The *Y*-axis represents the measured concentration from three sensors. Figure 6a–c shows the response of each sensor to its corresponding gas in three mixed gases, as S_1_ vs. methane, S_2_ vs. ethane, and S_3_ vs. propane, respectively. We can see from the figure that the sensors have deep cross-sensitivity to the other two gases. Therefore, it is difficult to recognize the concentration of each gas in this case.

**Figure 7:**
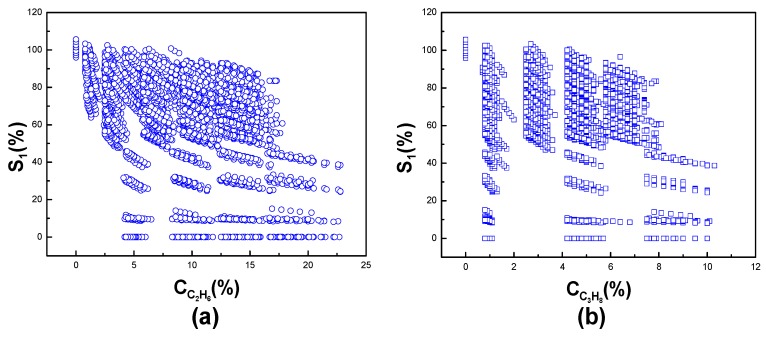
The cross-sensitivity of (**a**) ethane and (**b**) propane to methane.

Furthermore, the cross-sensitivities of ethane and propane to methane are shown in Figure 7a,b, respectively. Based on the facts we discussed above, it is concluded that the concentration of each gas in mixed gases cannot be read using the linear system. So, we use the MLP NN system to eliminate the cross-sensitivity.

**Figure 8:**
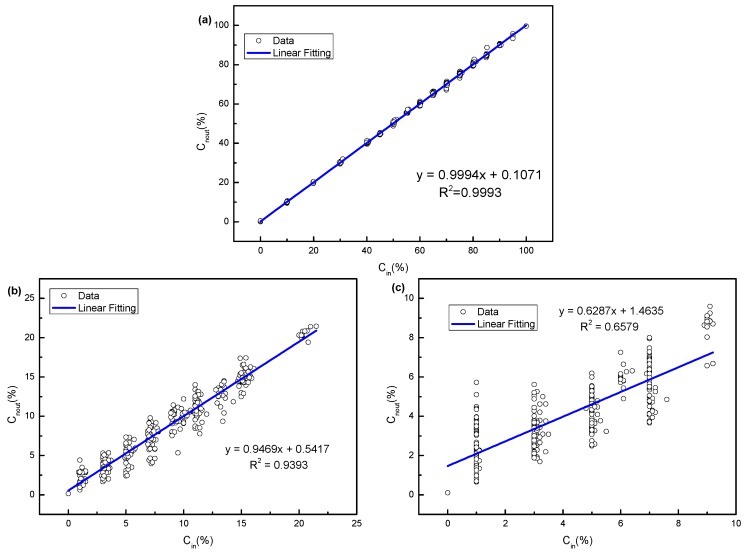
Results of the MLP NN algorithm for the recognition of (**a**) methane, (**b**) ethane and (**c**) propane, respectively.

In the MLP NN recognition algorithm, we use the linear regression model to evaluate the recognition accuracy of each gas. The difference between the output value of the recognition system and the input value set by the sample database is expressed by the coefficient of determination R^2^ (the expected value of R^2^ is 1). Figure 8 is a linear regression curve, which describes the recognition accuracy of methane, ethane and propane concentration, respectively. The horizontal axis is the real concentration of the sample database, and the vertical axis is the output concentration y of the recognition system. The coefficients of the determination of methane, ethane and propane were 0.9993, 0.9393 and 0.6579, respectively. The recognition accuracy of methane is higher than that of ethane and propane.

The mixed gas recognition results based on the MLP NN algorithm implemented by the FPGA are shown in Table 3, while each symbol used is explained in Table 2. The picture of the hardware circuit system is shown in Figure 4b, which corresponds to the diagram of circuit modules in Figure 4a. The synthesis results of the Electronic Design Automation (EDA) software can be listed as follows. The number of LE (logic elements) that were consumed by the NN algorithm module is 9894, the system clock is 50 MHZ, and the power consumption measured by the EDA power estimation function is 0.08 w. $C_{\mathrm{in}}$ in the first column represents the concentration of the mixed gas. Methane, ethane and propane constitute a mixture of three components. The total content of mixed gas is 100%. The concentration range of methane is 0~100%, and the concentration range of ethane and propane is 0~15%. Such a data sample library is built from the data of natural gas sources. $C_{\mathrm{sout}}$ in the second column represents the output values of the three sensors. It can be seen that in the mixture of gases, each sensor will be cross-sensitive to other gases, and cannot accurately respond with their corresponding gas concentration. The maximum error of S_1_ of methane is 12.3%, while that of S_2_ of ethane and S_3_ of propane is 72.3% and 84% respectively. $C_{\mathrm{corr}}$ in the third column represents the output value of the mixed gas recognition device based on the MLP NN algorithm implemented by the FPGA. 

We compare our work with similar works in Table 4, namely chromatography, single gas sensor and other ANN sensors. The advantage of this work is that we can recognize both the type and the concentration of each gas in mixed gases rather than separating mixed gases into individual gases by the chromatographic column and related methods.

In comparison, the output accuracy of methane, ethane and propane has been significantly improved, especially for propane whose R^2^ is 0.6579. The maximum recognition error of the three gases is reduced to 0.64%, 1.02% and 1.34%, respectively. The average recognition error of the three gases is reduced to 0.27%, 0.32% and 0.42%, respectively. The average error is less than 0.5% and meets users’ requirements. The computer provides the 64-bit double precision floating-point number and the effective digit is 15-bit. An FPGA can compute arbitrary precision operations, as long as one moves the decimal point. Since the FPGA uses integer digital computing, hardware recognition is at least four orders of magnitude faster than software training.

## 4. Conclusions

A mixed natural gas online testing device is developed to recognize the three main gases in natural gas by using an MLP NN and a BP algorithm implemented by an FPGA. MLP NN technology is adopted to reduce cross-sensitivity between mixture gases. MLP NN implementation reduces the maximum recognition error of the three gases to 0.64%, 1.02% and 1.34%, respectively. MLP NN FPGA implementation reduces the average recognition error of the three gases to 0.27%, 0.32% and 0.42%, respectively. FPGAs take advantage of parallel computing and greatly speed up the computational process of NNs. Within the range of 0–100%, the test errors for methane, ethane and propane are no more than 0.5%, and response speed is within several seconds.

## Figures and Tables

**Table 1:** Detailed information about our sensors.

Parameter	Value
Company	Dynament
Resolution	0.1%
Detection limit	0–200%
Selectivity	has cross-sensitivity to alkane
Response time	30 s

**Table 2:** Symbol illustrations.

Parameter	Illustration
$C_{\mathrm{in}}$	input concentration of each gas
$C_{\mathrm{sout}}$	gas sensor output
$S_{1}$	${\mathrm{CH}}_{4}$ sensor output
$S_{2}$	$C_{2}H_{6}\;$sensor output
$S_{3}$	$C_{3}H_{8}$ sensor output
$C_{\mathrm{nout}}$	NN output of gas concentration
$C_{\mathrm{corr}}$	final output of gas concentration

**Table 3:** Comparison of sensor output and recognition system output.

$C_{in}/\%$			$C_{sout}/\%$			$\mathrm{Error}\;\mathrm{of}\;C_{sout}/\%$			$C_{corr}/\%$			$\mathrm{Error}\;\mathrm{of}\;C_{corr}/\%$		
${\mathrm{CH}}_{4}$	$C_{2}H_{6}$	$C_{3}H_{8}$	$S_{1}$	$S_{2}$	$S_{3}$	$S_{1}$	$S_{2}$	$S_{3}$	${\mathrm{CH}}_{4}$	$C_{2}H_{6}$	$C_{3}H_{8}$	${\mathrm{CH}}_{4}$	$C_{2}H_{6}$	$C_{3}H_{8}$
0	5.5	3	0	11.2	9.3	0	5.7	6.3	0.11	5.47	3.35	0.11	0.03	0.35
9.8	5.5	3	8.8	19.0	16.9	1.0	13.5	13.9	9.66	5.34	2.98	0.14	0.16	0.02
20	4.9	6.4	27.8	31.4	29.2	7.8	26.5	22.8	20.1	5.92	5.06	0.1	1.02	1.34
30	8.3	5	40.8	29.8	27.8	10.8	21.5	22.8	29.4	8.68	4.83	0.6	0.38	0.17
40.1	3.2	5	51.3	32.8	30.7	11.2	29.6	25.7	40.2	3.48	4.98	0.1	0.28	0.02
50.3	11.5	5	62.6	83.8	84.4	12.3	72.3	79.4	50.7	11.8	4.27	0.4	0.3	0.43
59.9	13.4	1	70.9	84.3	85	11	70.9	84	60.2	13.1	0.57	0.3	0.3	0.43
70	5	3.2	78	51.9	52.3	8	46.9	49.1	69.8	4.76	3.2	0.2	0.24	0
80	5.1	5	84.9	68.1	69.2	4.9	63	64.2	79.9	5.53	4.02	0.1	0.43	0.98
90	3.2	4.8	90.9	65.5	66.1	0.9	62.3	61.3	90.3	3.27	4.4	0.3	0.07	0.4
100	0	0	100.8	21.1	20.2	0.8	21.1	20.2	99.5	0.28	0.21	0.5	0.28	0.21

**Table 4:** Comparison of our work with other related works.

Item	Chromatography	Single Gas Sensor	ANN Sensors	Our Work
Output form	spectral lines	electrical signal	electrical signal	electrical signal
Accuracy	high	low	high	high
Online	no	yes	yes	yes
Can be used for mixed gas	yes	no	no	yes
Can identify components	yes	no	no	yes
Response time	very slow	fast	fast	fast
Online communication	no	yes	yes	yes
